# Red blood cell distribution width to albumin ratio is associated with increased depression: the mediating role of atherogenic index of plasma

**DOI:** 10.3389/fpsyt.2025.1504123

**Published:** 2025-01-30

**Authors:** Tingting Shangguan, Jing Xu, Xiaochun Weng, Hao Lin

**Affiliations:** ^1^ Department of Intensive Care Unit, The Second Affiliated Hospital and Yuying Children's Hospital of Wenzhou Medical University, Wenzhou, Zhejiang, China; ^2^ Department of Endocrinology, The Second Affiliated Hospital and Yuying Children's Hospital of Wenzhou Medical University, Wenzhou, Zhejiang, China; ^3^ Department of Ultrasound, The Second Affiliated Hospital and Yuying Children's Hospital of Wenzhou Medical University, Wenzhou, Zhejiang, China; ^4^ Department of Gastroenterology, Pingyang Hospital of Wenzhou Medical University, Wenzhou, Zhejiang, China

**Keywords:** depression, albumin, red blood cell distribution width, cancer, inflammation

## Abstract

**Background:**

Recent studies have identified a correlation between inflammation and depression. This study aims to explore the correlation between the red blood cell distribution width (RDW) to albumin ratio (RAR), a practical measure for assessing inflammation, and depression in the general population.

**Methods:**

In this population-based cross-sectional study, data from 28932 adults aged≥18 years old in the NHANES during the period of 1999–2018 were analyzed. To examine the correlation between RAR and depression, multivariate logistic regression analyses, subgroup analyses, restricted cubic spline analyses, and interaction tests were conducted. Furthermore, a mediation analysis was performed to elucidate the role of atherogenic index of plasma (AIP) in mediating the effect of RAR on depression.

**Results:**

Multivariate logistic regression analyses and restricted cubic splines analysis indicated that RAR can exhibit a linearly correlation with depression (OR = 1.335; 95% CI: 1.222, 1.458). Subjects in RAR Q2, Q3, Q4 groups had an increased risk on depression as 22.8%, 22.9% and 51.9% than those in the Q1 group. This positive correlation was more pronounced in those with history of cancers. The ROC analysis indicated that the area under the curve (AUC) for RAR (AUC=0.593) was significantly greater than that for RDW and albumin individually. Mediation analysis indicated that AIP mediated 7.8% of the correlation of RAR with depression.

**Conclusions:**

The findings of this study indicated a significant linear positive correlation between RAR and the prevalence of depression, with AIP serving as a mediator.

## Introduction

Depression has been a widely acknowledged mental illness that has a substantial correlation with suicide. Approximately 40% of individuals died by suicide have been diagnosed as depression (1, 2). The World Health Organization (WHO) identified depression as the third largest contributor to the global disease burden as early as 2008 and predicted that it could become the leading cause of diseased burden by 2030 (3, 4). In the United States, it was estimated that 17.3 million adults, representing 6.7% of its population, experienced at least one major depressive episode in 2017, positioning it as the foremost cause of disability among individuals aged 15 to 44 (5, 6). Furthermore, depression is correlative with an increased risk of developing a range of physical illnesses, including cardiovascular disease, metabolic disorders, dementia, and cancers (7–10). Given the frequent comorbidity and high prevalence of depression with other diseases, preventing depression has become a critical public health concern.

A growing body of studies suggest that inflammation and nutrition state are considered as crucial mechanisms in depression (11, 12). RDW, a parameter derived from routine laboratory tests, serves as an indicator of the variability in erythrocyte volume. Increased RDW levels, which may arise from abnormal survival and impaired erythropoiesis of red blood cells, have been linked to a range of pathological conditions and increased chronic inflammation (13). Notably, the study revealed a correlation between increased RDW levels and depression. Serum albumin, the predominant circulating protein in the bloodstream, is a critical marker of both inflammatory response and nutritional status (14–17). The physiological functions of albumin encompass antioxidant, anti-inflammatory, antiplatelet aggregation properties, and anticoagulant, in addition to its role in maintaining colloid osmotic pressure (18). The literature provided evidence of a correlation between serum albumin levels and depression among various cohorts, including HIV patients, stroke survivors, individuals with chronic liver disease, elderly females, and community-dwelling populations (19–23).

Given the critical roles in diseases, physiological functions, and inflammation assessment, the combined evaluation of albumin concentration and RDW may provide valuable information for assessing the risk of depression. RAR, a parameter derived from standard laboratory tests, may provide the substantial information beyond individual values themselves. Recently, RAR has been identified as a potential risk biomarker for adverse outcomes in various diseases, including atrial fibrillation (24), acute myocardial infarction (25), heart failure (26), diabetes (27), stroke (28), and chronic kidney disease (29). However, the correlation between RAR and depression remains to be elucidated.

Consequently, this study aims to investigate the correlation between RAR and depression within a large, nationally representative sample of adults residing in data of Americans were sourced from the NHANES during the period of 1999-2018.

## Methods

### Research subjects and design

National Health and Nutrition Examination Survey (NHANES), conducted by National Center for Health Statistics (NCHS) (30), is a comprehensive study designed to assess the correlation between nutrition, health promotion, and disease prevention. The National Center for Health Statistics Ethics Review Board has approved the implementation of NHANES(NCHS ERB protocols #2011–17), and has obtained the written informed consent of all subjects following the Declaration of Helsinki. The survey shall be conducted every two years by taking physical examinations, interviews, and various sections covering dietary, demographic, examination, and laboratory data. Additional information regarding the NHANES database can be found at http://www.cdc.gov/nhanes.

**Figure 1:**
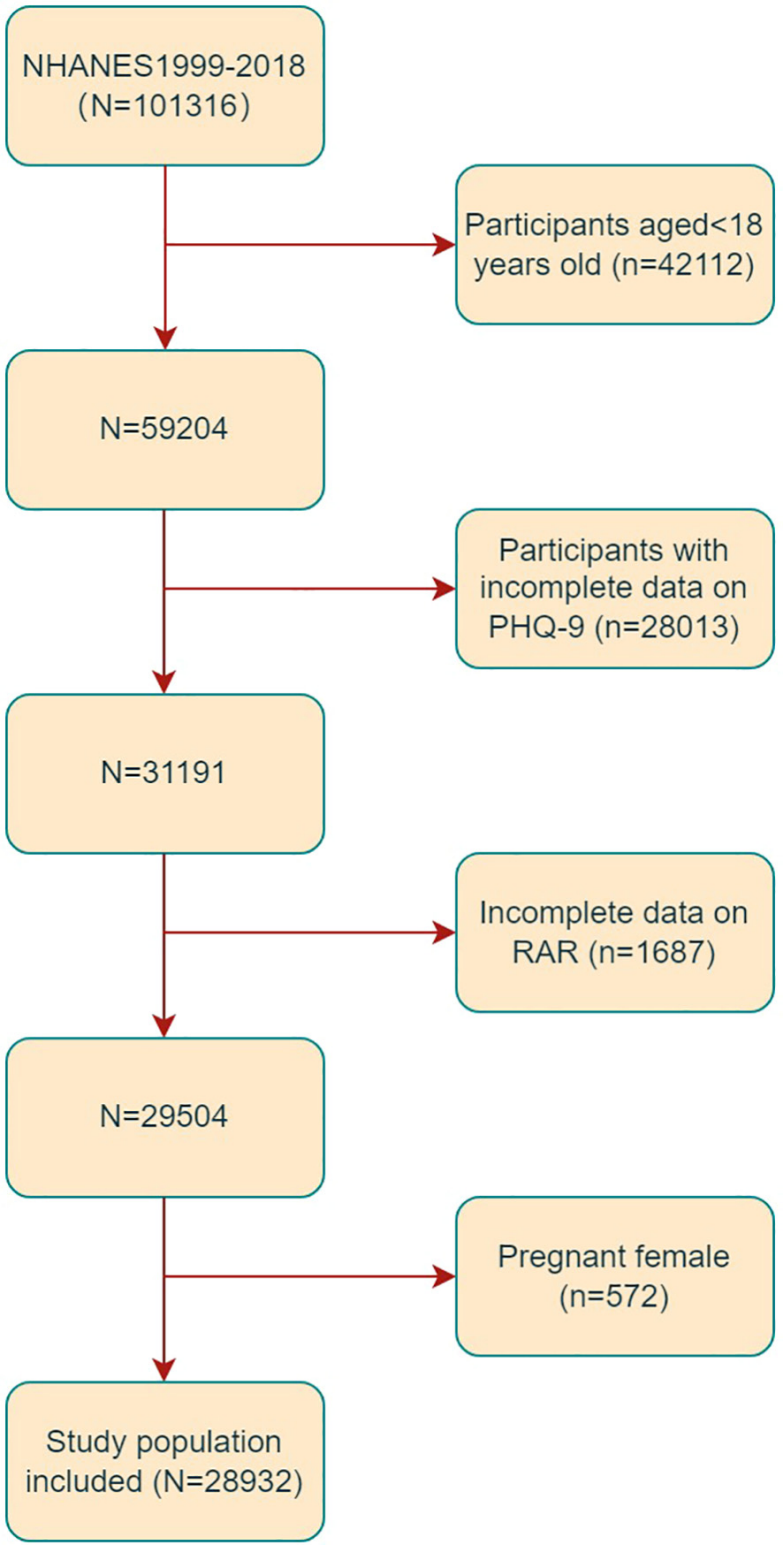
Flowchart of the sample selection from the 1999-2018 NHANES.

For the present study, data were collected from ten 2-year cycles of NHANES from 1999 to 2018. Subjects aged 18 years old or more were eligible for inclusion (n=59204). Subjects with incomplete Patient Health Questionnaire-9 (PHQ-9) (n=28013) and RAR (n=1687) data were excluded according to the exclusion criteria. Furthermore, 572 pregnant individuals were excluded (as shown in **Figure 1**). Ultimately, the study comprised a total of 28932 individuals.

### Ascertainment of depression

PHQ-9 (31, 32) is a widely utilized instrument for screening depression, consisting of 9 items with a scale from 0 to 3, resulting in a total score ranging from 0 to 27. A cumulative score of 10 or higher indicates depression. This threshold is frequently employed in epidemiological studies to identify individuals with depression and has been validated through clinical evaluation (31).

### Measurement of RAR

RAR was calculated as a previously established formula (33): RAR = RDW (%)/albumin (g/dL). Subjects were classified into four groups according to RAR quartiles as <2.82, 2.82–3.04, 3.04–3.31, and ≥3.31, with the lowest RAR category (<2.82) serving as the control group.

### Ascertainment of AIP

The determination of AIP was predicated upon the measurement on concentrations of high-density lipoprotein cholesterol (HDL-C) and triglycerides (TG) in the bloodstream. The precise mathematical formula utilized for calculating AIP was expressed as follows: log10 [TG (mmol/L)/HDL-C (mmol/L)] (34, 35).

### Covariates

Demographic and lifestyle data were collected through household interview questionnaires administered by highly trained medical personnel. Anthropometric measurements and biochemical parameters were obtained during medical examinations and subsequent laboratory assessments conducted at the Mobile Examination Center (MEC). The demographic data encompassed age, poverty-income ratio (PIR), gender, marital status, race, and education level. Self-reported health information included participants past medical history, specifically coronary heart disease (CHD), stroke, diabetes, hypertension, hyperlipidemia, and cancers, as well as details on physical activity, smoking, and alcohol consumption. Physical examination data, including body mass index (BMI), were recorded by trained health technicians at the MEC. Blood samples were collected at the MEC for the measurement of serum triglycerides (TG), urine albumin-to-creatinine ratio (UACR), total cholesterol (TC), red cell distribution width (RDW), high-density lipoprotein cholesterol (HDL-C), hemoglobin A1c (HbA1c), and low-density lipoprotein cholesterol (LDL-C). Less than 3% of values missed in total. Multiple imputation was performed for missing values. The definitions for alcohol intaking and smoking were consistent with previous reports (36). Estimated-glomerular filtration rate (eGFR) was calculated as the Chronic Kidney Disease Epidemiology Collaboration (CKD-EPI) formula (37, 38). Chronic kidney disease (CKD) was defined in accordance with current clinical guidelines as UACR exceeding 30 mg/g, eGFR of less than 60 mL/min/1.73 m², or both conditions (39, 40).

### Statistical analyses

In accordance with the PHQ-9 scores, subjects in this study were categorized into two distinct groups: depression and non-depression groups (31, 32). Normal distribution was evaluated by determining skewness with a Kolmogorov–Smirnov test. The normality of continuous variables was assessed and expressed as either median and interquartile range or mean ± SD. Between-group comparisons were made using Student’s t-test and the Kruskal–Wallis equality-of-populations rank test for normally distributed and skewed continuous variables, respectively, and using the χ2 test for categorical variables. To elucidate the correlation between RAR and depression, OR and 95% CI were estimated with logistic regression models. Variables demonstrating clinical and statistical significance in the univariate analyses (p<0.05) were incorporated into the multivariate analyses. Three models were employed in the analyses: Model 1 was unadjusted, and Model 2 was adjusted for gender and age. Model 3, the final multivariable model, included additional adjustments for race, hyperlipidemia, alcohol intaking, BMI, smoking, moderate physical activities, CHD, stroke, diabetes, CKD, PIR, education level, marital status, hypertension, HbA1c, and cancers. The dose–response correlation between depression and RAR was investigated through restricted cubic spline (RCS) curves, with a particular focus on potential non-linearity. Moreover, subgroup analyses were performed to ascertain whether the correlation between RAR and depression differed among subjects with varying characteristics. Subsequently, the mediation analyses were conducted with the mediation package, and the confidence interval of the mediation effect was evaluated with the Bootstrap method to quantify the proportion of AIP in the mediation effect. Data analyses were executed by using R software and Free Statistics software, with statistical significance established at a two-sided P value of less than 0.05.

## Results

### Baseline characteristics

**Table 1** shows a comprehensive summary of the subjects’ characteristics. Of the 28932 subjects included in the study, 14375 (49.7%) were females, with a mean age of 48.0 ± 18.7 years old. Among these subjects, 2503 (9.5%) presented with depression. The majority of individuals with depression were females, who had a higher prevalence of current or former smoking, lived alone, and had lower HDL-C and family PIR levels. Additionally, they were more likely to lack physical activities and had higher levels of BMI, RDW, HbA1c, TC, and TG. Furthermore, these individuals were more frequently correlative with underlying medical conditions such as hyperlipidemia, hypertension, CHD, diabetes, CKD, stroke, and cancers. RAR in the depression group was 3.26 ± 0.57, which was higher than 3.11 ± 0.46 observed in the non-depression group.

**Table 1:** Characteristics of the study population based on depression.

Characteristic	Total (n=28932)	PHQ-9<10 (n=26429)	PHQ-9≥10 (n=2503)	P value
Age	48.0 ± 18.7	48.0 ± 18.8	47.8 ± 16.9	0.759
Gender, %				< 0.001
Male	14557 (50.3)	13632 (51.6)	925 (37)	
Female	14375 (49.7)	12797 (48.4)	1578 (63)	
Race, %				< 0.001
Mexican American	4819 (16.7)	4416 (16.7)	403 (16.1)	
Other Hispanic	2788 (9.6)	2453 (9.3)	335 (13.4)	
Non-Hispanic White	12638 (43.7)	11583 (43.8)	1055 (42.1)	
Non-Hispanic Black	6022 (20.8)	5473 (20.7)	549 (21.9)	
Other Race	2665 (9.2)	2504 (9.5)	161 (6.4)	
Education level, %				< 0.001
Less than high school	6797 (25.0)	5917 (23.8)	880 (36.8)	
High school or above	20420 (75.0)	18909 (76.2)	1511 (63.2)	
Marital, %				< 0.001
Married/living with partner	17256 (62.3)	15988 (63.3)	1268 (52.5)	
Separated/divorced/widowed	5173 (18.7)	4530 (17.9)	643 (26.6)	
Never married	5248 (19.0)	4746 (18.8)	502 (20.8)	
Moderate physical activity, %				< 0.001
Yes	10007 (40.5)	9438 (42)	569 (25.3)	
No	14684 (59.5)	13008 (58)	1676 (74.7)	
Alcohol status, n%				0.949
Current or ever, %	19883 (71.1)	18149 (71.1)	1734 (71.2)	
Never	8083 (28.9)	7380 (28.9)	703 (28.8)	
Smoking status, n%				< 0.001
Current or ever, %	12539 (45.2)	11092 (43.9)	1447 (59.6)	
Never	15182 (54.8)	14203 (56.1)	979 (40.4)	
Hypertension, %				< 0.001
Yes	13242 (45.8)	11892 (45)	1350 (53.9)	
No	15690 (54.2)	14537 (55)	1153 (46.1)	
Diabetes, %				< 0.001
Yes	4072 (14.1)	3531 (13.4)	541 (21.7)	
No	24838 (85.9)	22881 (86.6)	1957 (78.3)	
Hyperlipidemia				< 0.001
Yes	20264 (70.0)	18364 (69.5)	1900 (75.9)	
No	8668 (30.0)	8065 (30.5)	603 (24.1)	
CHD, %				< 0.001
Yes	1107 (4.1)	954 (3.9)	153 (6.4)	
No	26024 (95.9)	23802 (96.1)	2222 (93.6)	
Stroke				<0.001
Yes	993 (3.7)	815 (3.3)	178 (7.5)	
No	26210 (96.3)	24003 (96.7)	2207 (92.5)	
CKD, %				< 0.001
Yes	5403 (18.7)	4820 (18.2)	583 (23.3)	
No	23529 (81.3)	21609 (81.8)	1920 (76.7)	
Cancer, %				0.002
Yes	2569 (9.4)	2302 (9.3)	267 (11.2)	
No	24640 (90.6)	22519 (90.7)	2121 (88.8)	
Body mass index, kg/m^2^	29.0 ± 6.9	28.8 ± 6.7	30.7 ± 8.3	< 0.001
HbA1c, %	5.72 ± 1.07	5.71 ± 1.05	5.89 ± 1.32	< 0.001
Albumin, g/dl	42.7± 3.4	42.7 ± 3.3	41.8 ± 3.6	< 0.001
Total cholesterol, mmol/L	4.86 (4.22, 5.61)	4.86 (4.22, 5.61)	4.94 (4.24, 5.72)	0.006
Triglycerides, mmol/L	1.15 (0.80, 1.71)	1.14 (0.79, 1.68)	1.31 (0.88, 1.94)	< 0.001
HDL-cholesterol, mmol/L	1.29 (1.06, 1.58)	1.29 (1.09, 1.60)	1.27 (1.03, 1.55)	< 0.001
LDL-cholesterol, mmol/L	2.85 (2.28, 3.46)	2.85 (2.28, 3.46)	2.90 (2.25, 3.54)	0.285
AIP	-0.05 ± 0.33	-0.06 ± 0.33	0.01 ± 0.35	< 0.001
PIR	2.50 ± 1.63	2.57 ± 1.63	1.69 ± 1.37	< 0.001
RDW	13.21 ± 1.35	13.18 ± 1.32	13.48 ± 1.58	< 0.001
RAR	3.12 ± 0.48	3.11 ± 0.46	3.26 ± 0.57	< 0.001

Values are mean±SD or number (%). P<0.05 was deemed significant. TC, total cholesterol; TG, triglyceride; HDL-c, High density lipoprotein cholesterol; LDL-c, Low density lipoprotein cholesterol; AIP, atherogenic index of plasma; PIR, poverty-income ratio; RAR, red blood cell distribution width to albumin ratio; CHD, coronary heart disease.

### Correlation between RAR and depression

**Figure 2:**
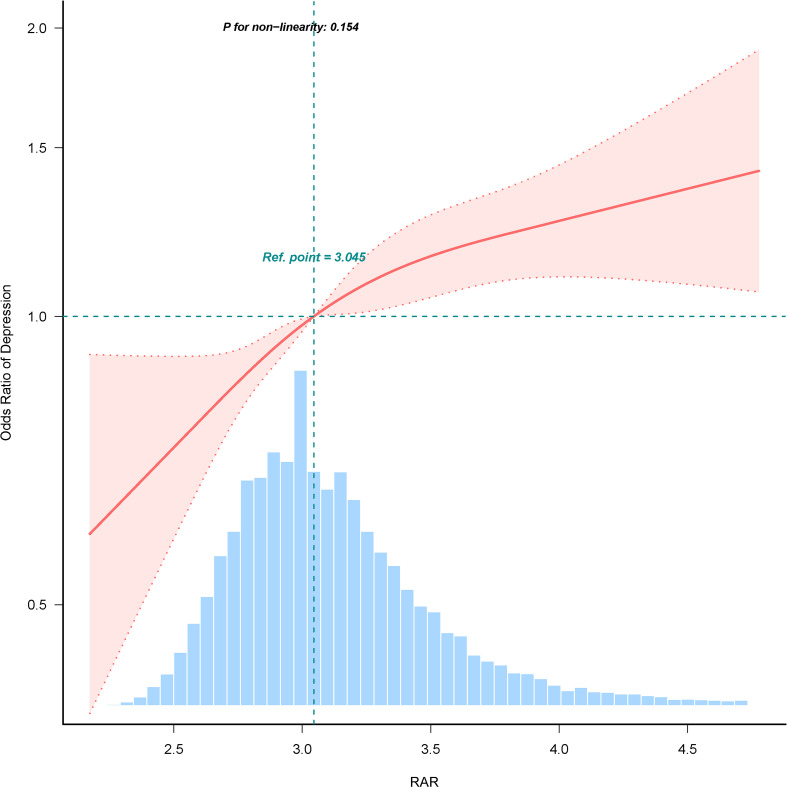
Restricted cubic spline fitting for the association between RAR and depression.

In multiple logistic regression analyses, a significant positive correlation between RAR and depression was identified after adjusting for confounders in Model 3 (OR=1.335, 95% CI: 1.222, 1.458). To further investigate RAR, these subjects were categorized into quartiles. In the totally adjusted Model 3, OR and 95% CI for subjects in RAR Q2, Q3, and Q4 groups were 1.228 (1.066, 1.415), 1.229 (1.065, 1.419), and 1.519 (1.311, 1.76), compared to the control group with Q1 group, respectively, for depression risk (*p* for trend <0.001), as detailed in **Table 2**. RCS analyses demonstrated a linear correlation between RAR and depression (as shown in **Figure 2**).

**Table 2:** Associations between RAR and depression.

Subgroups	Model1		Model2		Model3	
	OR (95%CI)	P-value	OR (95%CI)	P-value	OR (95%CI)	P-value
RAR	1.664 (1.550, 1.786)	<0.001	1.562 (1.450, 1.682)	<0.001	1.335 (1.222, 1.458)	<0.001
RAR (category)						
Q1	1(Ref)		1(Ref)		1(Ref)	
Q2	1.263 (1.109, 1.438)	4.00E-04	1.229 (1.078, 1.402)	0.0021	1.228 (1.066, 1.415)	0.0045
Q3	1.448 (1.276, 1.644)	<0.001	1.361 (1.193, 1.551)	<0.001	1.229 (1.065, 1.419)	0.0049
Q4	2.106 (1.869, 2.373)	<0.001	1.914 (1.685, 2.173)	<0.001	1.519 (1.311, 1.76)	<0.001
P for trend	1.276 (1.229, 1.325)	<0.001	1.236 (1.187, 1.286)	<0.001	1.134 (1.082, 1.187)	<0.001

Model 1: None covariates were adjusted; Model 2: gender and age were adjusted; Model 3, gender, age, race, hyperlipidemia, drinking, BMI, smoking, moderate physical activities, CHD, stroke, diabetes, CKD, PIR, hypertension, education level, marital status, HbA1c, and cancer were adjusted.

### Subgroup analysis

**Figure 3:**
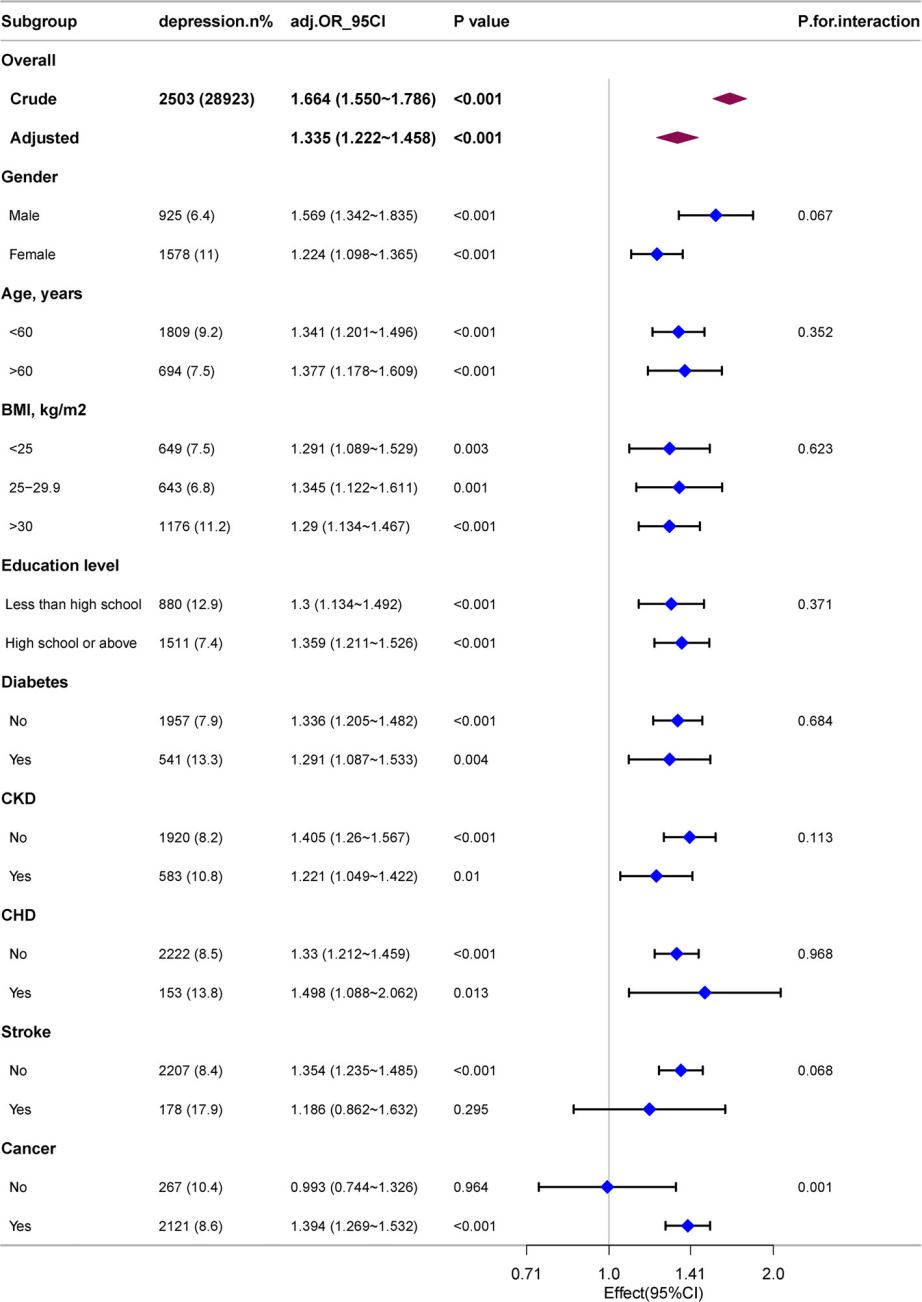
Association between RAR and the risk of depression in various subgroups.

Through subgroup analyses on further exploration of the correlation between RAR and depression across various populations stratified by age, BMI, education level, gender, and disease status (including CHD, diabetes, cancers, CKD and stroke), a significant interaction effect of cancer status on the correlation between RAR and depression was identified, with an interaction *p*<0.001. Specifically, among individuals with cancers, each one-unit increase in RAR was correlative with a 39.4% increase in the incidence of depression (OR: 1.394; 95%CI: 1.269–1.532). Notably, other covariates, including age and gender, did not demonstrate interactive effects on the correlation between RAR and depression (as shown in **Figure 3**).

### ROC analysis

**Figure 4:**
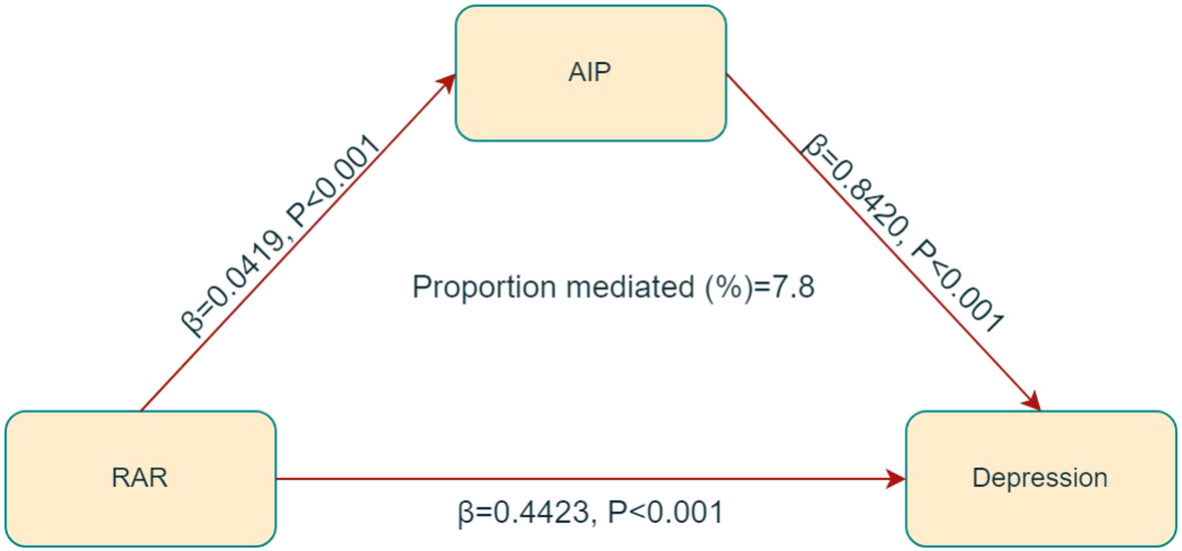
Mediated analysis model path diagram. RAR was defined as the independent variable; depression as the dependent variable; and AIP as the mediating variable.

ROC analyses revealed that, in subjects with depression, AUC for RAR was 0.593, which exceeded AUC for RDW (ACU=0.559) and albumin (AUC=0.575). These results were statistically significant (P < 0.05), as detailed in **Figure 4**.

### Mediation analysis

**Figure 5:**
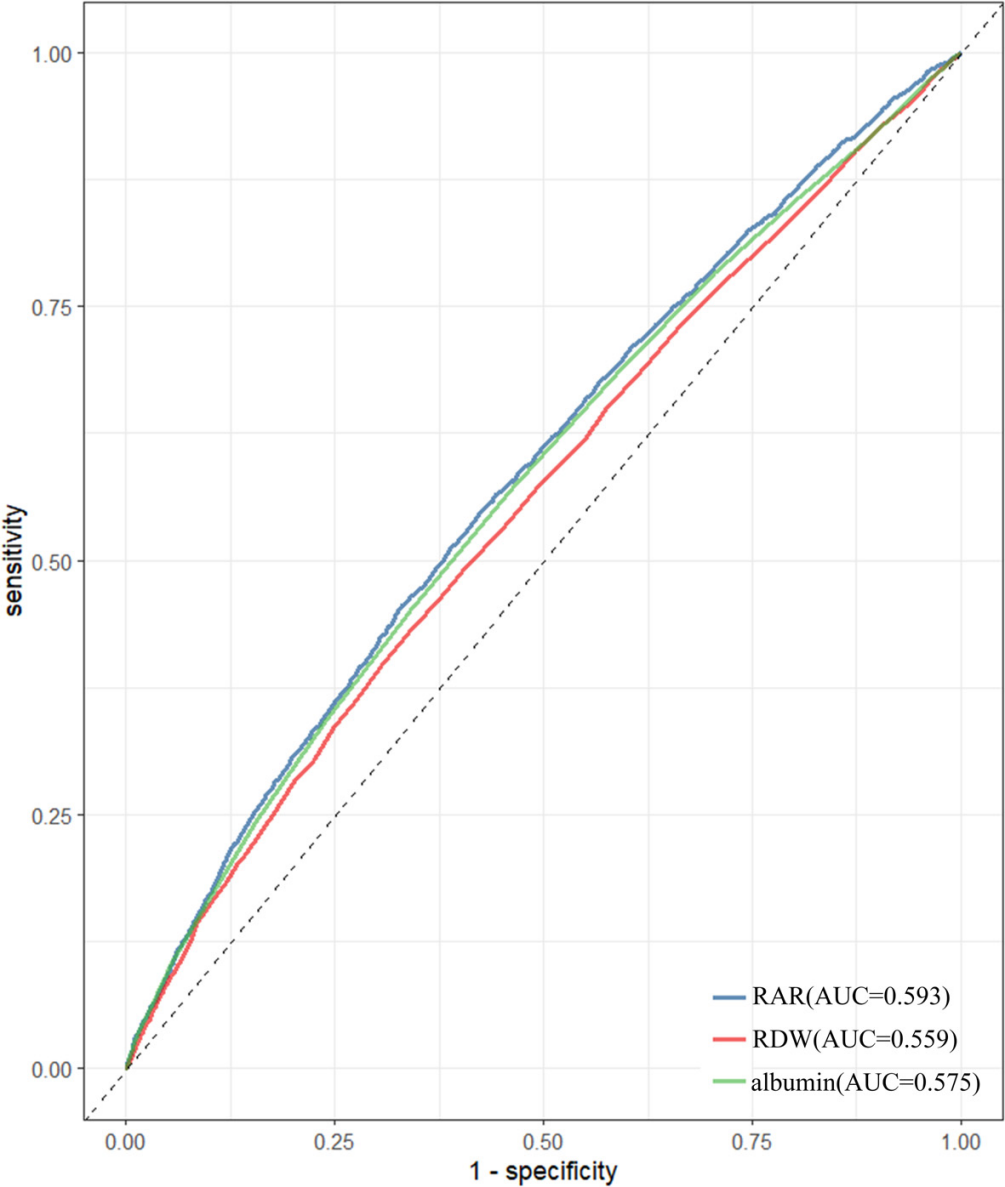
Receiver operating characteristic curves of RAR, RDW and albumin to identify depression.

In the mediation analysis, RAR was treated as the independent variable, depression as the dependent variable, and AIP as the mediator. The mediation model and its correlative pathways have been depicted in **Figure 5**. The results indicate a significant correlation between RAR and AIP (β = 0.0419, *p* < 0.001), as well as that between AIP and depression (β = 0.8420, *p* < 0.001). Further analyses revealed a significant indirect effect of RAR on depression through AIP, with an effect size of 0.0009 (*p* < 0.001), suggesting that AIP partially mediates the correlation between RAR and depression, accounting for approximately 7.8% of the total effect, as detailed in **Table 3**.

**Table 3:** Mediation analysis of AIP in the association between RAR and depression.

Independent variable	Mediator	Total effect		Indirect effect		Direct effect		Proportion mediated, %
		Coefficient (95% CI)	P value	Coefficient (95% CI)	P value	Coefficient (95% CI)	P value	
RAR	AIP	0.0015 (0.0104, 0.0122)	<0.001	0.0009 (0.0005, 0.0013)	<0.001	0.0106 (0.0097, 0.0113)	<0.001	7.8

## Discussion

This study identified a positive correlation between RAR and depression, with a notably stronger correlation among individuals with a history of cancers. RAR demonstrated superior diagnostic accuracy for depression compared to RDW and albumin independently. In addition, mediation analysis revealed that AIP partially mediated the correlation between RAR and depression.

In previous studies, it has been established that RAR possesses predictive capabilities regarding the mortality and severity of various cardiovascular disease, including stroke, acute coronary syndrome, coronary heart disease, heart failure, and atrial fibrillation (24–26, 28). Furthermore, an increased RAR has been linked to increased mortality rates in various conditions, including cancers, kidney diseases, chronic obstructive pulmonary disease, and diabetes (29, 41–43). Given that many of these conditions involve arteriosclerosis and injury, which are important risk factors for depression (44, 45), a strong correlation between RAR and depression was hypothesized. To the best of our knowledge, this is the first study to investigate the correlation between RAR and depression.

Erythrocyte pathologies and inflammation are often correlative. Inflammatory mediators contribute to endothelial and erythrocyte injury, thereby facilitating the development of atherosclerosis (46, 47). Erythrocyte dysfunction, in turn, can initiate inflammatory responses (47, 48) and oxidative dysregulation. Notably, RDW has been recognized as a biomarker for erythrocyte injury, which has been utilized in assessing depression risk (49, 50). Similarly, serum albumin can be recognized as an inflammatory marker (51). Previous studies have indicated that serum albumin levels are significantly lower in individuals with depressive disorders compared to healthy controls (23), and these levels are correlative with the severity of diseases (52, 53). Given that RDW and serum albumin are used to calculate RAR, this composite measure may provide a more reliable assessment of inflammation than either parameter alone. The results obtained from the ROC analysis of this study further affirmed the diagnostic potential of RAR for depression (as shown in **Figure 5**).

A significant interaction effect was identified between RAR and the history of cancers, indicating that the correlation between RAR and depression is particularly pronounced among individuals with cancers. This finding may be related to higher rates of depression among individuals with caners than those without cancers (54). Cancers are usually accompanied by chronic inflammation and malnutrition (55). The presence of cancers may exacerbate the effects of chronic inflammation correlative with increased RAR levels, potentially playing a contributory role in the onset and progression of depressive symptoms. Consequently, it is imperative to consider patients with cancers exhibiting high RAR levels as a high-risk group for depression. Further longitudinal and clinical studies are warranted to validate these findings.

The findings suggest that AIP partially mediates the correlation between inflammation (as determined by RAR) and depression, which underscores the importance of monitoring AIP in patients with a high RAR. Previous studies have demonstrated a significant correlation between depression and lipid metabolism disorders (56–59). Therefore, regulating AIP, particularly by reducing TG levels and increasing HDL-C levels, may mitigate the risk of depression in individuals with a high RAR.

The precise mechanisms underlying the correlation between depression and RAR remain incompletely elucidated. However, several potential pathways have been proposed. Chronic stress has been shown to induce sustained activation of microglia, which subsequently leads to the production of various proinflammatory cytokines, thereby contributing to the pathogenesis of depression (60). Additionally, early life stress can activate the hypothalamic-pituitary-adrenal (HPA) axis, resulting in increased cortisol levels. This hormonal imbalance can disrupt the homeostasis of neurotransmitters and the synthesis of nerve growth factors, ultimately leading to a decrease in hippocampal volume and the onset of depressive symptoms (61, 62). Furthermore, inflammation influences the release and reuptake of glutamate by glial cells. This dysregulation enables glutamate to bind to extra synaptic N-methyl-D-aspartate (NMDA) receptors, thereby inhibiting growth factors and induces excitotoxicity, and contributing to the development of depression (63). Serum albumin serves as the primary extracellular molecule for maintaining plasma redox homeostasis. A decrease in albumin may result in the dysregulation of oxidative stress, with increased levels of free radicals and oxidative injury being observable in patients with depression (64). Furthermore, albumin can be adversely affected by inflammatory processes as the predominant protein synthesized in liver, thereby indicating immune dysfunction and systemic inflammatory (65).

A significant strength of this study lies in the utilization of a nationally representative sample from the NHANES, enabling a thorough evaluation of diverse characteristics in population. This methodological approach enhances the generalizability of findings to a broader spectrum of American adults. Furthermore, the study meticulously controlled for numerous confounding variables, yielding robust estimates of the independent correlation between RAR and the prevalence of depression. Finally, an intermediary analysis was performed in this study to explore the correlation between chronic inflammation, AIP, and depression.

It is essential to recognize several significant limitations inherent in this study. Firstly, the cross-sectional research design constrains the capacity to infer causality between RAR and depression. To address this limitation, future research should incorporate experimental methodologies and longitudinal surveys to elucidate temporal correlation and underlying mechanisms. Secondly, the diagnosis of depression by using the PHQ-9 scale relies on self-reported data and lacks validation by clinical practitioners. It is important to acknowledge that PHQ-9 is extensively employed in both epidemiological and clinical contexts, which has been rigorously validated, demonstrating the high specificity and the high sensitivity (66). Lastly, the findings are specific to Americans, thereby constraining the generalizability of the results to other demographic groups.

## Conclusion

In summary, a significant correlation between RAR levels and depression after adjusting for multiple confounding variables was identified in this study. Increased RAR levels may have important implications for the treatment and diagnosis of depression. Nevertheless, this hypothesis necessitates further validation through prospective studies.

## Data Availability

Publicly available datasets were analyzed in this study. This data can be found here: NHANES, http://www.cdc.gov/nhanes.
